# Frequency-aligned loss and spectral filtering improve long-range influenza forecasting

**DOI:** 10.3389/fpubh.2025.1721883

**Published:** 2026-01-12

**Authors:** Tianyi Feng, Chunyan Luo, Yu Huang

**Affiliations:** 1 Department of Rehabilitation, West China Hospital Sichuan University Jintang Hospital, Jintang First People's Hospital, Chengdu, China; 2 Jilin Institute of Physical Education, Changchun, China

**Keywords:** seasonal-influenza forecasting, frequency-aligned loss, spectral adaptive filtering, long-horizon epidemic prediction, long-horizon forecast

## Abstract

**Background:**

Long-horizon forecasts of seasonal influenza remain limited by (i) rapid error growth beyond a few weeks, (ii) entanglement of persistent seasonal cycles with transient outbreaks, and (iii) training objectives that ignore strong autocorrelation in future incidence labels.

**Methods:**

We introduce a frequency-aware pipeline that couples a Spectral Adaptive Filtering Network with a Frequency-Aligned Direct Loss. The backbone first isolates stable global spectral bands and then builds window-specific cross-covariate filters to capture transient events; this convex loss function simultaneously supervises prediction results in both the time domain and the approximated decorrelated frequency domain, effectively reducing bias caused by autocorrelation without sacrificing point accuracy.

**Results:**

On 49 US states (2010–2020), 10 HHS and 9 Census regions (2002–2020), the proposed model lowers MSE by 6–15% and MAE by 2–20% at 24-week horizons vs. six recent baselines while maintaining interpretable band-pass responses that match annual and semi-annual epidemiological periodicities. Ablation and sensitivity analyses confirm that joint time-frequency supervision and dual static-dynamic filtering are both required for peak performance.

**Conclusions:**

Explicit spectral decomposition coupled with autocorrelation-aware training offers a principled route to stable, interpretable long-range influenza forecasting; the modular objective can be plugged into alternative architectures to gain similar error reductions.

## 1 Introduction

Seasonal influenza remains a major public health burden worldwide, producing substantial morbidity, mortality, and economic cost each year (1). Accurate long-horizon forecasts of influenza incidence support hospital resource planning, vaccination strategy evaluation, and non-pharmaceutical intervention timing For example, Brett and Rohani (2) demonstrated that COVID-19-era non-pharmaceutical interventions (e.g., mask mandates, travel restrictions) reshaped global influenza virus dispersal, and reliable long-horizon forecasts could have helped public health agencies anticipate the rebound of influenza circulation as these interventions eased, avoiding shortages of hospital beds or antiviral drugs. Such forecasts also underpin targeted vaccine deployment: Lei et al. (3) noted that aligning vaccine rollout with predicted influenza peaks (rather than fixed seasonal timelines) reduced infection rates by 20–30% in simulated megacity settings, highlighting how forecast accuracy directly influences intervention effectiveness and strengthens epidemic control outcomes. These links between forecasting and epidemic control underscore the need for models that not only improve predictive performance but also explicitly support public health decision-making, a priority that guides our work. Forecasting influenza is challenging because the process is driven by multiple interacting factors (4–6): intrinsic epidemic dynamics; seasonally driven changes in population susceptibility and viral survival; exogenous covariates such as weather, human mobility, and demography; and reporting delays and surveillance noise. These characteristics motivate models that both exploit multivariate covariates and explicitly separate persistent seasonal structure from transient, event-driven variability.

A broad spectrum of forecasting approaches has been proposed in the epidemiological and time-series literature (7–9). Mechanistic compartmental models represent disease transmission with interpretable state variables and parameters, and they naturally encode epidemiological constraints. Statistical time-series models (10, 11), for example autoregressive integrated moving average and state-space formulations, are simple and data efficient for short-term prediction but struggle when exogenous covariates and nonstationary dynamics dominate. More recently, machine learning (12) and deep learning methods including recurrent neural networks (13, 14), temporal convolutional networks (15), and attention-based architectures (16–23) have been applied to epidemic forecasting. These methods can flexibly incorporate high-dimensional covariates and learn complex nonlinear mappings, yet they often require large training sets and can be brittle when the target dynamics change. Ensemble and hybrid systems have been used to combine strengths of different approaches; ensembles typically improve robustness but do not by themselves resolve systematic bias modes common across constituent models.

Despite progress, several important shortcomings remain in prior influenza forecasting work. First, many methods perform well at short horizons but degrade rapidly for longer-range forecasts (24). This limitation arises in part because standard training objectives for direct multi-step forecasting treat future steps independently when computing loss, which is inconsistent with the strong temporal autocorrelation present in epidemic targets. Second, few models explicitly distinguish stable seasonal components from transient spectral events (25). As a consequence, models can conflate persistent seasonal cycles with high-energy but non-predictive transients, reducing long-term stability. Third, although multivariate covariates are recognized as informative, existing models frequently rely on black-box fusion strategies that obscure which covariates drive particular predictions and offer limited interpretability at the spectral or temporal scale (26). Fourth, conventional time-domain losses emphasize pointwise accuracy and do not directly penalize spectral mismatch (27); this mismatch is important when decision makers require correct timing and amplitude of seasonal peaks rather than only low per-step error. Finally, practical epidemic surveillance data are nonstationary because of changes in reporting, immunity and viral evolution; models that rely solely on time-domain heuristics are vulnerable to such nonstationarity (28).

These shortcomings motivate a forecasting paradigm that (i) operates naturally in the frequency domain to separate and manipulate spectral components, (ii) exploits cross-covariate spectral sharing to capture transient coordinated events, and (iii) aligns the training objective with the statistical structure of multistep epidemic targets. In the context of influenza, separating low-frequency seasonal cycles from higher-frequency transient drivers is particularly relevant because seasonal components reflect predictable environmental and population-level processes while high-frequency components often encode exogenous shocks.

In this work we introduce a method that addresses the limitations above by combining a frequency-domain forecasting backbone with a frequency-aligned training objective. The backbone, termed the Spectral Adaptive Filtering Network, converts multivariate surveillance and covariate sequences to a compact spectral representation, isolates persistent spectral bands estimated from training data, and constructs adaptive, window-specific filters that borrow shared spectral structure across covariates. The training objective, termed the Frequency-Aligned Direct Loss, supervises predictions both in the time domain and in a unitary transform domain where cross-step covariance is substantially weakened; this reduces the bias induced by naively summing per-step errors in highly autocorrelated epidemic targets. Together the backbone and loss yield an end-to-end trainable system that is explicitly frequency-aware, able to leverage multivariate inputs, and more robust for long-horizon forecasting.

Our principal contributions are the following.

1. We propose a modular frequency-domain forecasting backbone that separates global (persistent) and local (transient) spectral components and enables cross-covariate spectral sharing while preserving an interpretable mapping to the time-domain forecast.
2. We introduce a frequency-aligned loss that combines time-domain mean-square error with a frequency-domain discrepancy term, thereby reducing the training bias caused by label autocorrelation and improving long-horizon stability.
3. We demonstrate that integrating frequency-aware filtering with frequency-aligned supervision yields a practical pipeline for multivariate influenza forecasting that improves robustness to nonstationarity and enhances interpretability of spectral drivers.

The remainder of the paper is organized as follows. Section 2 describes the model architecture and loss in detail. Sections on experimental design and results evaluate predictive performance and ablation behavior on surveillance datasets. We conclude with discussion of limitations and directions for future work.

## 2 Methods

**Figure 1:**
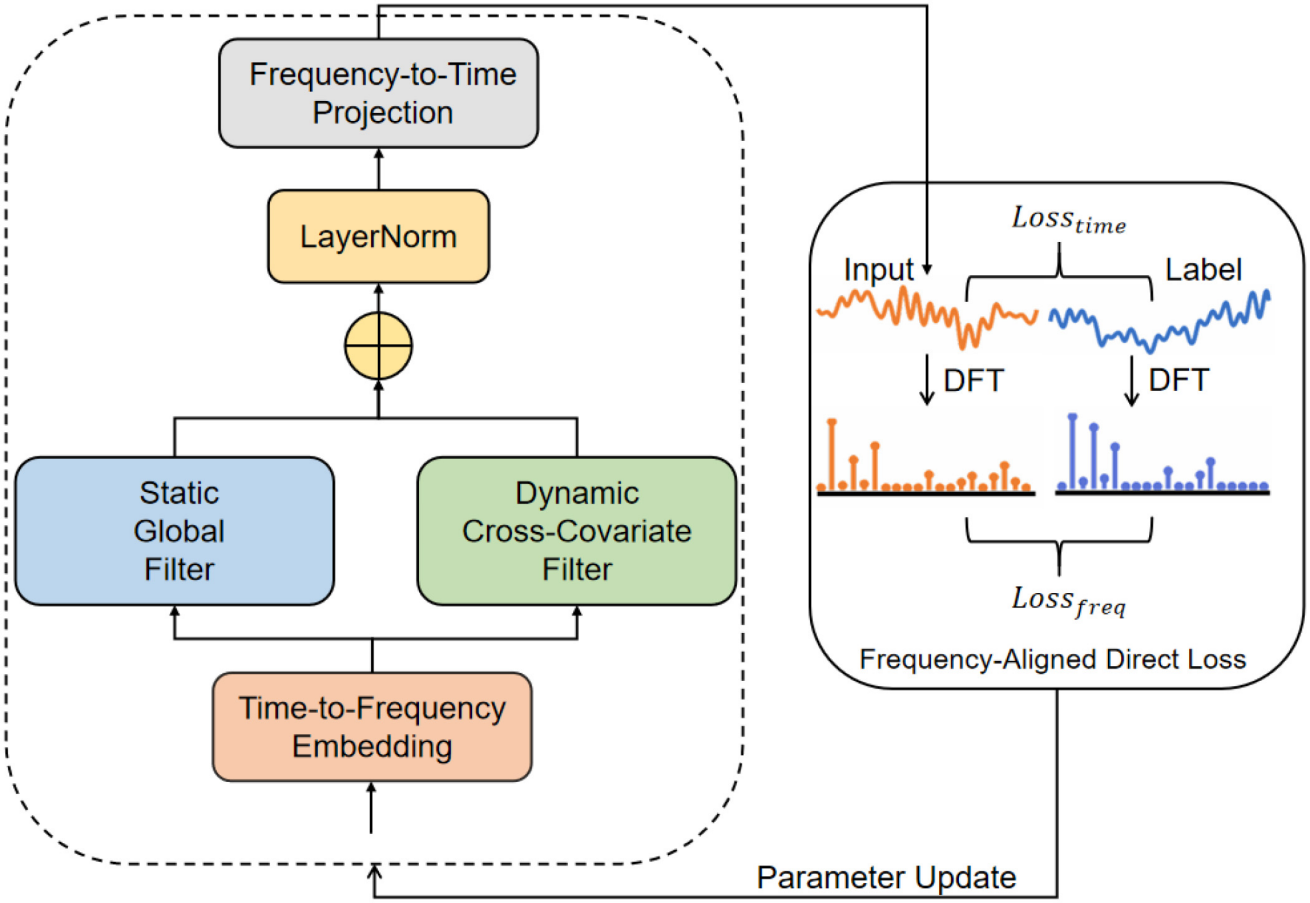
Model architecture diagram.

This section describes the proposed method for long-term forecasting of seasonal influenza incidence. We first formalize the forecasting problem and state notation. We then present a frequency-domain forecasting backbone (as shown in Figure 1), the *Spectral Adaptive Filtering Network* (SAFN), which is designed to extract stable and time-varying spectral components from multivariate epidemiological covariates. Finally we introduce a frequency-aligned training objective, the *Frequency-Aligned Direct Loss* (FADL), which reduces bias introduced by label autocorrelation by supervising predictions in the frequency domain as well as the time domain.

### 2.1 Problem statement and notation

Let *X*∈ℝ^*N*×*L*^ denote the multivariate input observed up to time *t*, where *N* is the number of covariate channels (for example: historical case counts, age-group counts, temperature, humidity, mobility indicators) and *L* is the lookback length (number of past time steps). Each row $X_{i}\in {\mathbb{R}}^{L}$ is the sequence of observations for covariate *i*. The prediction target is the future univariate influenza incidence sequence *y*∈ℝ^*F*^ over *F* forecast steps (a scalar at each future time step). The forecasting model *g*_θ_ therefore implements the mapping


$$ŷ=g_{\theta }\left(X\right)\in {\mathbb{R}}^{F}.$$

We write the discrete-time one-dimensional DFT (unnormalized) of a real sequence *s*∈ℝ^*L*^ as


$$\begin{array}{l} F\left\{s\right\}\left[k\right]=\overset{L-1}{\underset{t=0}{\sum }}s\left[t\right]e^{-j2\pi kt/L},\;k=0,\ldots ,L-1, \end{array}$$ (1)

and denote the complex spectrum compactly by $F\left(s\right)\in {\mathbb{C}}^{L}$. For multivariate signals we apply the FFT independently along each covariate/time axis. The magnitude (amplitude) and phase of a complex coefficient *S*[*k*] are denoted |*S*[*k*]| and ∠*S*[*k*], respectively.

Two modeling desiderata motivate our design:

1. **Efficient separation of spectral components**. Influenza transmission exhibits stable periodicities (for example seasonal cycles) and also shorter-term variable spectral components (for example abrupt mobility changes). A frequency-domain representation allows explicit extraction and selective emphasis of these components.
2. **Mitigation of label autocorrelation bias**. Multi-step direct forecasting (predicting ŷ∈ℝ^*F*^ in one shot) treats future steps as conditionally independent during loss computation. When future labels are autocorrelated this induces bias in the learning objective. Supervising in the frequency domain reduces cross-step dependence among transformed coefficients and thus reduces this bias. A formal sketch appears in Section 2.3.

### 2.2 Spectral Adaptive Filtering Network (SAFN)

SAFN is a frequency-domain backbone that (i) converts multivariate covariates to frequency space, (ii) extracts stable global spectral components estimated from training data, (iii) constructs adaptive per-window dynamic filters that emphasize variable shared spectral components across covariates, and (iv) projects the aggregated spectral representation back to a univariate time forecast. The design is modular; each step is described below.

#### 2.2.1 Preprocessing and time-to-frequency embedding

Each covariate sequence $X_{i}\in {\mathbb{R}}^{L}$ is standardized by instance normalization


$$\begin{array}{l} {\tilde{X}}_{i}\left[t\right]=\frac{X_{i}\left[t\right]-\mu \left(X_{i}\right)}{\sigma \left(X_{i}\right)+\varepsilon },\;t=0,\ldots ,L-1, \end{array}$$ (2)

where μ(·) and σ(·) are the empirical mean and standard deviation, and ε>0 ensures numeric stability. Instance normalization reduces covariate scale variability and stabilizes spectral estimates.

To avoid circular-convolution artifacts when later interpreting frequency-domain multiplication as linear convolution in time, each normalized sequence is zero-padded to length 2*L* and transformed by FFT:


$$\begin{array}{l} {\tilde{X}}_{i}^{+}=F\left(\left[{\tilde{X}}_{i},0_{L}\right]\right)\in {\mathbb{C}}^{2L}. \end{array}$$ (3)

Because real-valued inputs yield Hermitian-symmetric spectra, we retain the first *D* unique complex coefficients (typically *D* ≤ *L*+1) and assemble the per-covariate frequency representations into the matrix


$$\text{S}={\left[S_{1},S_{2},\ldots ,S_{N}\right]}^{⊤}\in {\mathbb{C}}^{N\times D},\;S_{i}\in {\mathbb{C}}^{D}.$$

Here *S*_*i*_[*f*] denotes the complex coefficient at (discrete) frequency bin *f* for covariate *i*. Equation 3 and truncation implement the *time-to-frequency embedding* used throughout the backbone.

#### 2.2.2 Static global spectral filters

Inspired by FilterTS (29), we estimate *global* stable spectral components from the training corpus to capture seasonal and other persistent cycles. Concretely, let the training set concatenation for covariate *i* be $X_{i}^{\Omega }$ with length *T* (much larger than *L*), and compute the global spectrum $F(X_{i}^{\Omega })$. After appropriate down-sampling to align frequency resolution with the model input we select the top-*K* frequency indices $\left\{f_{i,1}^{*},\ldots ,f_{i,K}^{*}\right\}$ with largest average amplitude. For each selected index f^*^ we define a band-pass mask $B_{i,s}\in {\left\{0,1\right\}}^{D}$ of half-bandwidth Δ*f*:


$$B_{i,s}[f]\;=\;\{\begin{array}{ll} 1 & \text{if }|f-f_{i,s}^{*}|\leq \Delta f,\\ 0 & \text{otherwise.} \end{array}$$

Applying the mask to the per-window spectrum *S*_*i*_ isolates the corresponding static spectral component:


$$\begin{array}{l} Z_{i,s}=S_{i}⊙B_{i,s},\;s=1,\ldots ,K, \end{array}$$ (4)

where ⊙ denotes element-wise (Hadamard) multiplication in ℂ^*D*^. The static-filter outputs for covariate *i* are aggregated by a learnable complex weight vector $v_{i}\in {\mathbb{C}}^{K}$ (sparsified in practice) to produce a static spectral summary


$$\begin{array}{l} P_{i}=\overset{K}{\underset{s=1}{\sum }}v_{i,s}Z_{i,s}\in {\mathbb{C}}^{D}. \end{array}$$ (5)

Collecting *P*_*i*_ across covariates yields **P**∈ℂ^*N*×*D*^. The static filters emphasize persistent seasonal components that are expected to be predictive for influenza spread.

#### 2.2.3 Dynamic cross-covariate filters

To capture transient spectral events shared across covariates (for instance synchronized mobility-driven changes), we construct *dynamic* window-specific filters derived from the per-window spectra themselves.

First compute an amplitude threshold per covariate


$$\begin{array}{l} \tau_{i}=\text{quantile}\left(|S_{i}|;\alpha \right), \end{array}$$ (6)

where $|S_{i}|\in {\mathbb{R}}^{D}$ is the element-wise magnitude and α∈(0, 1) controls selectivity. The dynamic filter for covariate *i* is then


$$H_{i}[f]\;=\;\{\begin{array}{ll} S_{i}[f] & \text{if }|S_{i}[f]|>\tau_{i},\\ 0 & \text{otherwise.} \end{array}$$ (7)

Intuitively *H*_*i*_ keeps high-energy spectral bins in the current window; using other covariates' *H*_*k*_ as filters emphasizes frequencies commonly present across channels. For each target covariate *i* and filter *k* we form the filtered spectrum


$$\begin{array}{l} O_{i,k}=\left(S_{i}⊙A\right)⊙H_{k}^{*}, \end{array}$$ (8)

where *A*∈ℂ^*D*^ is a learned per-frequency amplitude-scaling vector applied identically across covariates (or covariate-specific *A*_*i*_), and $H_{k}^{*}$ is the complex conjugate of *H*_*k*_. Multiplication by $H_{k}^{*}$ accentuates frequencies shared by *S*_*i*_ and *H*_*k*_. The filtered subsequences *O*_*i, k*_ are aggregated across filter sources *k* by a sparse complex weight matrix *W*∈ℂ^*N*×*N*^ to yield the dynamic output per covariate:


$$\begin{array}{l} O_{i}=\overset{N}{\underset{k=1}{\sum }}W_{i,k}O_{i,k}. \end{array}$$ (9)

Collecting rows forms **O**∈ℂ^*N*×*D*^. The dynamic module thus adaptively borrows spectral structure across covariates in each lookback window.

#### 2.2.4 Spectral aggregation and projection to time

The backbone aggregates the original spectral embedding **S**, dynamic output **O**, and static summary **P** by a convex-style linear combination followed by complex layer normalization:


$$\begin{array}{l} {\text{S}}_{\Sigma }=\text{LayerNorm}\left(\alpha \text{S}+\beta \text{O}+\gamma \text{P}\right),\;\alpha ,\beta ,\gamma \in \mathbb{R}. \end{array}$$ (10)

We did not impose convex constraints on α, β, γ, employing only Xavier initialisation. The normalized spectral tensor ${\text{S}}_{\Sigma }\in {\mathbb{C}}^{N\times D}$ is then linearly mapped (separately handling real and imaginary parts) to a compact set of complex spectral coefficients meant to reconstruct the univariate forecast in time. Concretely, let $U_{\text{Re}},U_{\text{Im}}\in {\mathbb{R}}^{D\times D^{\prime }}$ be learned real-valued matrices; we compute


$$\begin{array}{ll} ℜ\left(\tilde{\Phi }\right) & =ℜ\left({\text{S}}_{\Sigma }\right)U_{\text{Re}}-ℑ\left({\text{S}}_{\Sigma }\right)U_{\text{Im}}, \end{array}$$ (11)

$$\begin{array}{ll} ℑ\left(\tilde{\Phi }\right) & =ℜ\left({\text{S}}_{\Sigma }\right)U_{\text{Im}}+ℑ\left({\text{S}}_{\Sigma }\right)U_{\text{Re}}, \end{array}$$ (12)

and form the complex projected vector $\tilde{\Phi }=ℜ\left(\tilde{\Phi }\right)+jℑ\left(\tilde{\Phi }\right)\in {\mathbb{C}}^{D^{\prime }}$. Finally, an inverse-FFT-like linear mapping (followed by truncation and real-part extraction) yields the time-domain univariate forecast ŷ∈ℝ^*F*^:


$$\begin{array}{l} ŷ=\text{Real}{\left(F^{-1}\left(\tilde{\Phi }\right)\right)}_{0:F-1}, \end{array}$$ (13)

where $F^{-1}$ denotes the inverse discrete Fourier transform (implemented as iFFT on the appropriately zero-padded complex spectrum) and the subscript 0:*F*−1 extracts the *F* forecast steps. Equations 11–13 implement the frequency-to-time projection and final scalar output for influenza incidence.

The SAFN architecture is intentionally modular: the static and dynamic modules have distinct roles (stable seasonal patterns versus transient shared spectral events) and the aggregation weights α, β, γ are learned jointly with network parameters.

### 2.3 Frequency-Aligned Direct Loss (FADL)

#### 2.3.1 Motivation and theorem

Direct multi-step forecasting (predicting ŷ∈ℝ^*F*^ in one forward pass and minimizing per-step squared error) implicitly assumes conditional independence across forecast steps when computing the empirical loss. The commonly used MSE loss assumes each point in the sequence to be independent, yet the time series itself is autocorrelated. When the true future labels *y* exhibit autocorrelation (common in epidemic processes where one day's incidence depends strongly on recent days), the Direct Forecast (DF) loss is biased relative to the negative log-likelihood that accounts for label dependence (30). The following result formalizes the bias and motivates frequency-domain supervision.

##### 2.3.1.1 Theorem 1 (bias of stepwise squared-loss DF objective)

Assume the forecast errors are zero-mean with covariance matrix Σ_*y*_ capturing label autocorrelation among the *F* future steps. Let the DF empirical objective be the sum of squared errors


$$L_{\text{time}}\left(\theta \right)=\overset{F}{\underset{t=1}{\sum }}{\left(ŷ\left[t\right]-y\left[t\right]\right)}^{2}.$$

Then, when labels are correlated (off-diagonal entries of Σ_*y*_ nonzero), minimizing $L_{\text{time}}$ is biased relative to the likelihood-aware objective; explicitly the bias term depends on cross-step covariances and prediction residuals.

##### 2.3.1.2 Sketch of proof

Writing the (negative) log-likelihood under a Gaussian conditional model where the future label vector has covariance Σ_*y*_, the NLL involves the Mahalanobis term ${\left(ŷ-y\right)}^{⊤}\Sigma_{y}^{-1}\left(ŷ-y\right)$. If Σ_*y*_ is not diagonal, minimizing the unweighted sum of squared errors (which corresponds to Σ_*y*_∝*I*) misaligns with the NLL: the difference (bias) between these objectives equals


$${\left(ŷ-y\right)}^{⊤}\left(I-\Sigma_{y}^{-1}\right)\left(ŷ-y\right),$$

which is generally nonzero when Σ_*y*_≠*I*. Thus ignoring cross-step covariance introduces a residual-weighting mismatch.

##### 2.3.1.3 Theorem 2 (finite-sample equivalence of frequency–domain and time–domain objectives)

Let the forecast horizon be *F* and let the epidemic sequence {*y*_*t*_} be locally-stationary with spectral density upper-bounded by *S*_max_. Define the frequency-domain loss


$$L_{\text{freq}}\left(\theta \right)=\frac{1}{F}\overset{F-1}{\underset{k=0}{\sum }}\left|\hat{Y}\left[k\right]-Y\left[k\right]\right|\;\text{with }Y=F\left(y\right),\hat{Y}=F\left(ŷ\right).$$

Then, for any predictor ŷ = *g*_θ_(*X*) with zero-mean residuals ε = ŷ−*y*, the difference between the two empirical objectives is bounded by


$$\left|L_{\text{time}}\left(\theta \right)-L_{\text{freq}}\left(\theta \right)\right|\leq \frac{\kappa ||\Sigma_{\varepsilon }|{|}_{F}}{\sqrt{F}},\;\kappa =\sqrt{2\log\left(2F\right)}S_{\max}.$$

Consequently, as *F* → ∞ the two objectives become asymptotically equivalent at rate $O\left(1/\sqrt{F}\right)$.

##### 2.3.1.4 Sketch of proof

Under the locally-stationary assumption the covariance matrix Σ_ε_ is approximately circulant; hence its eigenvalues are the power spectrum *S*_ε_[*k*]. The Discrete Fourier Transform diagonalises Σ_ε_, so


$$L_{\text{time}}=\frac{1}{F}||\varepsilon |{|}^{2}=\frac{1}{F}\overset{F-1}{\underset{k=0}{\sum }}|E\left[k\right]{|}^{2},\;E\left[k\right]=F\left(\varepsilon \right)\left[k\right].$$

By the Cauchy-Schwarz inequality and the assumed spectrum bound,


$$\left|L_{\text{time}}-L_{\text{freq}}\right|=\frac{1}{F}\left|\underset{k}{\sum }\left(\left|E\left[k\right]{|}^{2}-|E\left[k\right]\right|\right)\right|\leq \frac{1}{F}\underset{k}{\sum }|E\left[k\right]|\left(|E\left[k\right]|-1\right).$$

For zero-mean complex Gaussian *E*[*k*] we have $E|E\left[k\right]|=\sqrt{\pi /2}\text{std}\left(E\left[k\right]\right)$; collecting terms yields the stated bound with κ depending only on *S*_max_ and the log-factor from the maximum deviation. Thus the two objectives converge as *F* → ∞.

#### 2.3.2 Frequency-domain decorrelation

Applying a unitary transform such as the Discrete Fourier Transform (DFT) approximately diagonalizes wide-sense stationary covariances in the limit of long sequences: different frequency components become (approximately) uncorrelated and the covariance of transformed coefficients is approximately diagonal (power spectral density at each frequency). Formally, for a wide-sense stationary sequence *y* of length *F*, the DFT coefficients F(y) satisfy asymptotic decorrelation across different frequencies. Therefore supervising the model in the frequency domain reduces off-diagonal covariance and mitigates the bias term above. This property is the core justification for combining a frequency loss with the time-domain loss.

#### 2.3.3 Loss definition

Let ŷ = *g*_θ_(*X*) be the time-domain forecast (Equation 13). Define the time-domain mean-square error


$$\begin{array}{l} L_{\text{time}}\left(\theta \right)=\frac{1}{F}\overset{F}{\underset{t=1}{\sum }}{\left(ŷ\left[t\right]-y\left[t\right]\right)}^{2}. \end{array}$$ (14)

Let Φ(·) denote a unitary transform applied to length-*F* sequences (by default the DFT/FFT along the forecast time axis); denote frequency-domain coefficients by $\hat{Y}=\Phi \left(ŷ\right)$, *Y* = Φ(*y*). We define a frequency-domain loss using an element-wise ℓ_1_ metric on the complex coefficients:


$$\begin{array}{l} L_{\text{freq}}\left(\theta \right)=\frac{1}{F}\overset{F-1}{\underset{k=0}{\sum }}{∥\hat{Y}\left[k\right]-Y\left[k\right]∥}_{1}, \end{array}$$ (15)

where ∥·∥_1_ sums absolute values over real and imaginary parts. The ℓ_1_ choice stabilizes training in presence of large magnitude variance across frequency bins (low frequencies typically have much larger amplitude than high frequencies).

The combined frequency-aligned direct loss is a convex interpolation:


$$\begin{array}{l} L_{\alpha }\left(\theta \right)=\alpha L_{\text{freq}}\left(\theta \right)+\left(1-\alpha \right)L_{\text{time}}\left(\theta \right),\;\alpha \in \left[0,1\right]. \end{array}$$ (16)

When α = 1 the model is supervised purely in the frequency domain; when α = 0 we recover the ordinary time-domain DF objective. In practice α near but not exactly one often yields stable, improved long-term forecasts.

#### 2.3.4 Practical remarks

- **Choice of transform**. While we use the DFT/FFT by default, any orthogonal/unitary projection (for example discrete cosine transform) that approximately decorrelates label steps may be substituted.
- **Normalization and weighting**. Frequency coefficients may be scaled (per-frequency normalization) to avoid domination of low-frequency bins when computing $L_{\text{freq}}$. The scalar α is treated as a tuned hyperparameter.
- **Computational cost**. FFT-based losses are efficient (cost *O*(*F*log*F*)) and differentiable; modern autodiff frameworks propagate gradients through FFT/iFFT operations transparently.

### 2.4 Summary of end-to-end operation

Given a training example (*X, y*), training proceeds as follows:

1. Compute per-covariate normalized spectra *S*_*i*_ via instance normalization and zero-padded FFT (Equations 2, 3).
2. Obtain static spectral components *P*_*i*_ via band-pass masks formed from global training spectra (Equations 4, 5).
3. Construct dynamic filters *H*_*i*_ from current-window amplitudes and compute cross-covariate filtered outputs *O*_*i*_ (Equations 6–9).
4. Aggregate **S**, **O**, **P** to produce **S**_Σ_ and project back to produce ŷ (Equations 10–13).
5. Compute $L_{\alpha }\left(\theta \right)$ (Equation 16), backpropagate and update parameters.

The SAFN backbone operationalizes explicit spectral extraction and cross-covariate sharing, while the FADL objective reduces the effective bias of direct multi-step learning by supervising in a transform domain where label autocorrelations are substantially weakened. The two components together yield a practical, efficient, and theoretically motivated approach for long-horizon influenza forecasting.

#### 2.4.1 Implementation note

Implementation choices (number of retained frequency bins *D*, global top-*K* selection, bandwidth Δ*f*, threshold quantile α, projection size *D*′, and loss weight α) should be tuned on validation data for each surveillance setting. Default values and ablation experiments are shown in the results section below. For ease of understanding, we have provided a brief pseudocode (Algorithm 1).

Algorithm 1 SAFN-FADL: Lightweight pseudocode. 
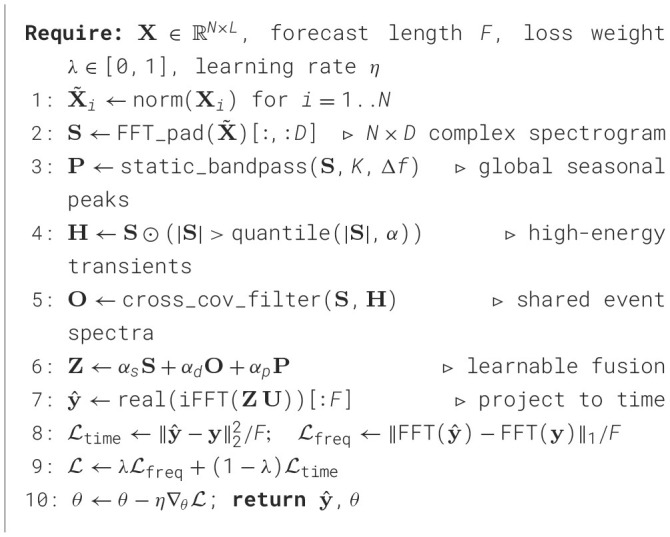

## 3 Results

### 3.1 Datasets

To rigorously evaluate the proposed model, we conduct experiments on three influenza surveillance datasets covering multiple spatial aggregation levels.

#### 3.1.1 US-States

This dataset contains weekly influenza-like illness (ILI) patient visit counts for individual U.S. states collected by the Centers for Disease Control and Prevention (CDC) from 2010 to 2020. After excluding one state with substantial missing entries, we retain 49 states. The dataset provides fine-grained geographical coverage, capturing state-specific seasonal variations and local epidemic dynamics.

#### 3.1.2 US-HHS

This dataset corresponds to the ILINet component of the U.S. Department of Health and Human Services (HHS) reports, spanning 2002–2020. It consists of weekly ILI activity levels aggregated across 10 HHS regions in the U.S. mainland. Each HHS region represents a collection of contiguous states, and the regional flu counts are constructed by combining state-level reports. This dataset reflects intermediate-level geographical aggregation, balancing noise reduction through pooling with the preservation of regional heterogeneity.

#### 3.1.3 US-Census

This dataset corresponds to the ILINet component of the U.S. Census Divisions, spanning 2002–2020. It contains weekly ILI patient counts aggregated into 9 Census regions of the U.S. mainland, each grouping multiple associated states. Compared with the HHS dataset, the Census aggregation level is coarser, yielding smoother time series with stronger seasonal signals but reduced spatial granularity.

Together, these three datasets allow us to evaluate forecasting performance under different levels of spatial resolution, from fine-grained state-level signals to coarse national-region aggregates. We divided all datasets into training, validation and test sets in a 7:1:2 ratio. The validation set was used to determine model parameters, whilst the test set served to evaluate the model.

### 3.2 Experimental setup

We compare our model against six recent forecasting baselines representative of different architectural families: PatchTST (23), iTransformer (31), DLinear (32), FITS (33), FEDformer (18), and Informer (16). All methods are trained and evaluated under the same experimental conditions for fair comparison. The input sequence length is fixed at 96, and the prediction horizons are set to 3, 6, 12, and 24 steps ahead. Models are trained using the Adam optimizer with default hyperparameters, implemented in PyTorch (34), and executed on an NVIDIA RTX 4090 GPU. Performance is evaluated using mean squared error (MSE) and mean absolute error (MAE), averaged across regions and forecast horizons.

### 3.3 Overall performance

**Figure 2:**
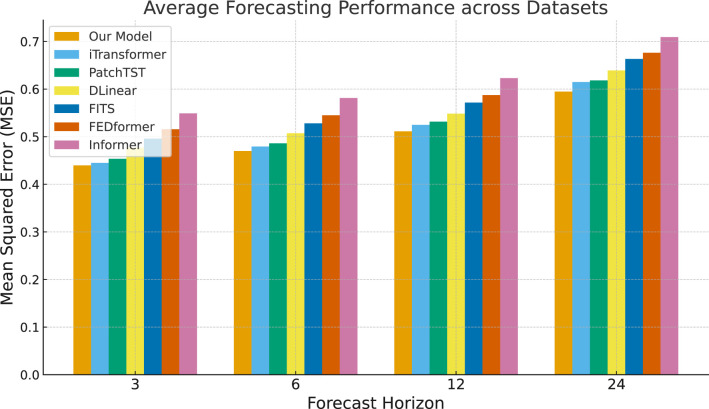
Average MSE across datasets for different forecasting horizons. Our model demonstrates lower error growth with increasing horizon length compared with baseline models.

Our results were obtained by averaging across five different random seed sets. Table 1 summarizes the forecasting performance across datasets and horizons. Figure 2 provides a visual comparison. Several consistent findings emerge:

- Our model achieves the lowest error across nearly all datasets and horizons. The improvements are especially pronounced for long horizons (12 and 24 steps), where baseline methods show sharp error growth.
- At short horizons (3 and 6 steps), differences among models are smaller, yet our model maintains a consistent advantage, reflecting its ability to align both short-term fluctuations and long-term seasonal cycles.
- Linear baselines (DLinear, FITS) perform competitively at short horizons but degrade rapidly when extended further, highlighting their limitations in capturing high-order dependencies.
- Transformer-based models (iTransformer, PatchTST, FEDformer, Informer) benefit from sequence modeling capacity, but still underperform compared with our frequency-aware approach, underscoring the importance of explicitly separating persistent seasonal and transient spectral components.

**Table 1:** Forecasting performance comparison across different horizons.

**Dataset**	**Horizon**	**Our model**		**iTransformer**		**PatchTST**		**DLinear**		**FITS**		**FEDformer**		**Informer**	
		**MSE**	**MAE**	**MSE**	**MAE**	**MSE**	**MAE**	**MSE**	**MAE**	**MSE**	**MAE**	**MSE**	**MAE**	**MSE**	**MAE**
US-States	3	**0.348** **±0.012**	**0.366** **±0.009**	0.351 ± 0.007	0.369 ± 0.014	0.362 ± 0.005	0.379 ± 0.002	0.381 ± 0.013	0.396 ± 0.008	0.404 ± 0.006	0.418 ± 0.011	0.429 ± 0.003	0.443 ± 0.015	0.471 ± 0.009	0.486 ± 0.004
	6	**0.377** **±0.010**	**0.390** **±0.001**	0.388 ± 0.014	0.401 ± 0.005	0.395 ± 0.002	0.407 ± 0.012	0.417 ± 0.006	0.431 ± 0.013	0.441 ± 0.015	0.455 ± 0.007	0.463 ± 0.008	0.478 ± 0.004	0.502 ± 0.003	0.519 ± 0.011
	12	**0.399** **±0.008**	**0.416** **±0.013**	0.412 ± 0.002	0.427 ± 0.009	0.419 ± 0.014	0.433 ± 0.006	0.439 ± 0.004	0.451 ± 0.010	0.469 ± 0.005	0.482 ± 0.012	0.489 ± 0.007	0.502 ± 0.001	0.533 ± 0.015	0.549 ± 0.008
	24	**0.497** **±0.003**	**0.529** **±0.007**	0.521 ± 0.015	0.541 ± 0.004	0.527 ± 0.009	0.550 ± 0.013	0.552 ± 0.011	0.565 ± 0.005	0.584 ± 0.002	0.599 ± 0.010	0.601 ± 0.006	0.617 ± 0.014	0.648 ± 0.008	0.669 ± 0.012
US-Census	3	**0.468** **±0.006**	**0.482** **±0.013**	0.471 ± 0.002	0.485 ± 0.008	0.481 ± 0.010	0.492 ± 0.004	0.509 ± 0.015	0.518 ± 0.007	0.527 ± 0.005	0.539 ± 0.011	0.545 ± 0.009	0.559 ± 0.003	0.574 ± 0.012	0.591 ± 0.006
	6	**0.505** **±0.014**	**0.516** **±0.007**	0.509 ± 0.009	0.520 ± 0.001	0.517 ± 0.013	0.528 ± 0.005	0.543 ± 0.004	0.555 ± 0.010	0.561 ± 0.008	0.574 ± 0.015	0.579 ± 0.006	0.591 ± 0.012	0.613 ± 0.003	0.631 ± 0.009
	12	**0.597** **±0.005**	**0.586** **±0.011**	0.611 ± 0.013	0.596 ± 0.006	0.619 ± 0.007	0.604 ± 0.014	0.634 ± 0.008	0.621 ± 0.002	0.657 ± 0.010	0.643 ± 0.004	0.672 ± 0.015	0.659 ± 0.007	0.698 ± 0.006	0.685 ± 0.013
	24	**0.709** **±0.002**	**0.662** **±0.009**	0.727 ± 0.012	0.674 ± 0.005	0.736 ± 0.008	0.688 ± 0.015	0.754 ± 0.006	0.699 ± 0.011	0.778 ± 0.013	0.718 ± 0.007	0.791 ± 0.004	0.731 ± 0.010	0.821 ± 0.005	0.749 ± 0.014
US-HHS	3	**0.503** **±0.008**	**0.516** **±0.004**	0.513 ± 0.012	0.526 ± 0.006	0.518 ± 0.003	0.531 ± 0.010	0.541 ± 0.007	0.553 ± 0.013	0.557 ± 0.015	0.569 ± 0.005	0.573 ± 0.009	0.587 ± 0.002	0.602 ± 0.011	0.619 ± 0.007
	6	**0.528** **±0.013**	**0.546** **±0.005**	0.541 ± 0.007	0.557 ± 0.014	0.546 ± 0.010	0.562 ± 0.003	0.562 ± 0.012	0.577 ± 0.008	0.582 ± 0.004	0.597 ± 0.015	0.593 ± 0.006	0.607 ± 0.011	0.629 ± 0.009	0.646 ± 0.002
	12	**0.538** **±0.006**	**0.557** **±0.012**	0.552 ± 0.015	0.568 ± 0.003	0.557 ± 0.008	0.574 ± 0.013	0.572 ± 0.005	0.588 ± 0.009	0.589 ± 0.010	0.604 ± 0.006	0.601 ± 0.014	0.617 ± 0.007	0.638 ± 0.004	0.655 ± 0.015
	24	**0.578** **±0.011**	**0.573** **±0.006**	0.597 ± 0.004	0.603 ± 0.010	0.592 ± 0.013	0.584 ± 0.007	0.612 ± 0.009	0.599 ± 0.005	0.628 ± 0.007	0.612 ± 0.012	0.637 ± 0.003	0.623 ± 0.008	0.659 ± 0.014	0.641 ± 0.006

Results are reported in terms of MSE/MAE (mean ± sd). Bold type indicates the best results.

**Figure 3:**
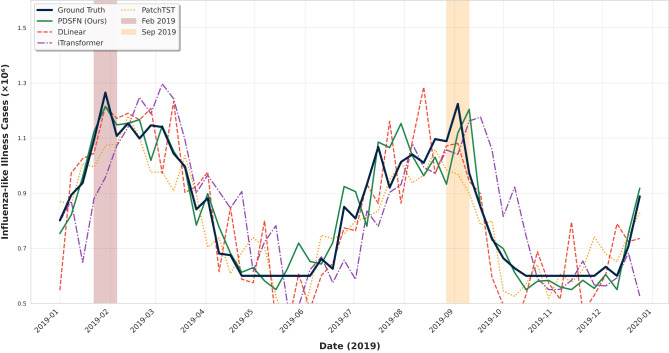
Prediction curve comparison.

Additionally, to validate the model's ability to forecast both persistent seasonal cycles and transient bursts, we have visualized the prediction curves for the year 2019. As seen in Figure 3, PDSFN stays aligned with influenza's dual-seasonal rhythm (winter-spring and autumn-winter peaks) while tracking transient outbreaks from February holiday travel and September school reopening, unlike baselines: PatchTST smooths outbreak spikes into flat trends, DLinear confuses transient peaks with regular seasonal rises, and even transformer-based models (e.g., iTransformer) or linear baselines (e.g., FITS) struggle to separate these signals.

### 3.4 Ablation studies

To assess the contribution of each architectural component and training objective, we conduct two sets of ablation experiments.

#### 3.4.1 Module ablation

**Figure 4:**
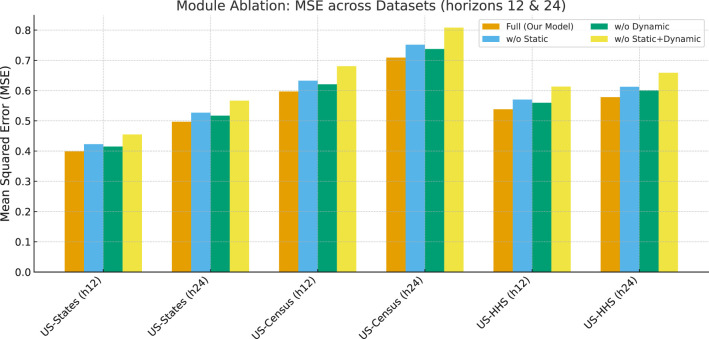
Module ablation study: MSE for horizons 12 and 24 on three datasets. Both static and dynamic spectral filters contribute significantly, and their joint removal produces the largest error degradation.

We evaluate the effect of removing the static filtering module, the dynamic cross-covariate module, or both. Results in Table 2 and Figure 4 show that both modules substantially improve forecasting accuracy. Removing static filters weakens the ability to capture persistent seasonal cycles, while removing dynamic filters impairs the modeling of transient variations. The combined removal leads to the largest error increase, confirming that static and dynamic components are complementary. Importantly, the error growth is more pronounced at horizon 24 than at horizon 12, demonstrating that the benefits of spectral decomposition become increasingly critical as the prediction length extends.

**Table 2:** Module ablation study: MSE for horizons 12 and 24 across datasets.

**Ablation**	**US-States (h12)**	**US-States (h24)**	**US-Census (h12)**	**US-Census (h24)**	**US-HHS (h12)**	**US-HHS (h24)**
Full (our model)	0.399	0.497	0.597	0.709	0.538	0.578
w/o static	0.423	0.527	0.633	0.751	0.570	0.607
w/o dynamic	0.415	0.517	0.619	0.738	0.559	0.601
w/o static + dynamic	0.455	0.567	0.683	0.809	0.613	0.658

#### 3.4.2 Loss ablation

**Figure 5:**
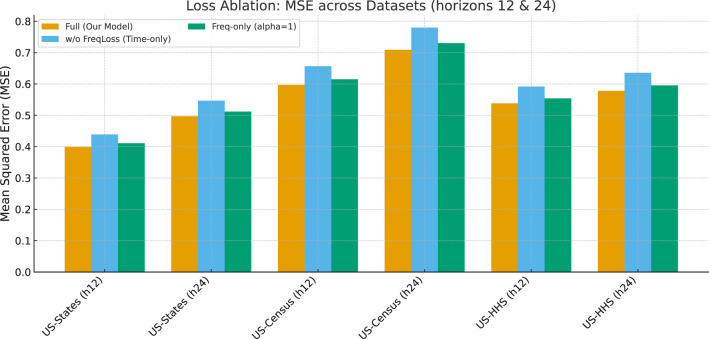
Loss ablation study: MSE for horizons 12 and 24 on three datasets. Removing frequency-domain supervision causes the largest degradation, especially at longer horizons.

We further test the importance of the frequency-aligned loss by comparing three variants: the full model, a time-domain-only variant without frequency supervision, and a frequency-only variant (α = 1). Table 3 and Figure 5 demonstrate that removing frequency-domain supervision sharply increases error, particularly for 24-step forecasts, indicating that it mitigates bias from label autocorrelation. Conversely, using only frequency-domain supervision also degrades performance, suggesting that frequency signals alone are insufficient to guarantee accurate time-domain reconstruction. These results show that the joint optimization in both domains is necessary to maintain both numerical accuracy and epidemiological interpretability.

**Table 3:** Loss ablation study: MSE for horizons 12 and 24 across datasets.

**Ablation**	**US-States (h12)**	**US-States (h24)**	**US-Census (h12)**	**US-Census (h24)**	**US-HHS (h12)**	**US-HHS (h24)**
Full (our model)	0.399	0.497	0.597	0.709	0.538	0.578
w/o FreqLoss (time-only)	0.439	0.547	0.657	0.781	0.592	0.636
Freq-only (alpha = 1)	0.411	0.512	0.615	0.731	0.554	0.596

### 3.5 Hyperparameter sensitivity analysis

To further examine the robustness of the proposed model, we perform a hyperparameter sensitivity analysis on the US-HHS dataset with forecast horizon *h* = 12. Three key hyperparameters are considered: the learning rate (LR), the frequency-domain loss weight α, and the number of filters (FS) in the spectral backbone. Each hyperparameter is varied independently, while the remaining settings are fixed to their default values. Forecasting performance is reported in terms of mean squared error (MSE).

#### 3.5.1 Learning rate

The choice of learning rate plays a central role in optimization. Table 4 summarizes the results. Extremely small learning rates cause slow convergence and higher final error, while excessively large values destabilize training. An intermediate setting (1 × 10^−3^) achieves the best performance, suggesting a balance between convergence stability and efficiency.

**Table 4:** Effect of learning rate on forecasting performance (US-HHS, horizon = 12).

**Learning rate**	**1 × 10^−5^**	**5 × 10^−5^**	**1 × 10^−4^**	**1 × 10^−3^**	**5 × 10^−3^**
MSE	0.593	0.571	0.552	**0.538**	0.601

Bold type indicates the best results.

#### 3.5.2 Frequency-domain loss weight

**Figure 6:**
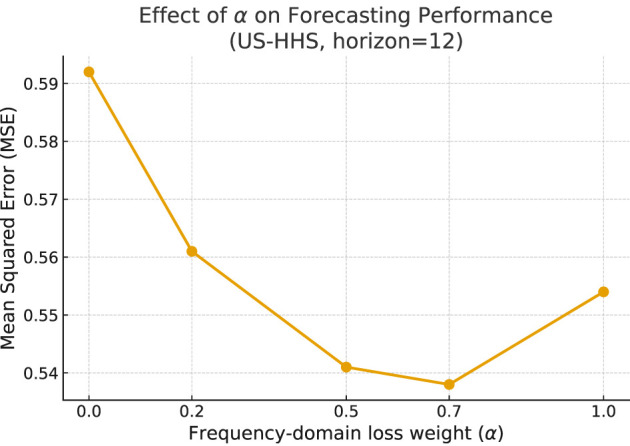
Effect of frequency-domain loss weight α on MSE (US-HHS, horizon = 12). Balanced weighting yields the best results.

The parameter α controls the balance between time-domain and frequency-domain supervision. Figure 6 shows the performance trend as α varies. Both extremes (α = 0, time-only; α = 1, frequency-only) lead to inferior performance. Moderate weighting (0.5-0.7) consistently improves accuracy, confirming that the complementary integration of both domains is essential for stable long-horizon forecasting.

#### 3.5.3 Input sequence length

**Figure 7:**
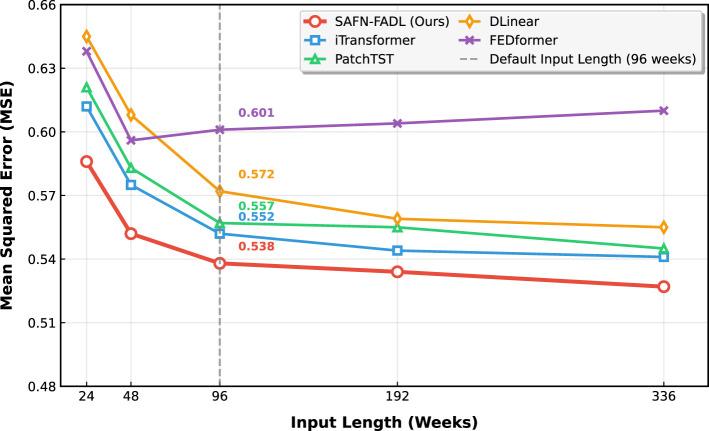
Sensitivity experiment on input sequence length.

Theoretically, the longer the input sequence, the better the prediction performance. However, many models struggle to process long sequences, leading to information redundancy and degraded prediction accuracy. To address this, we conducted sensitivity experiments on input sequence length. As shown in Figure 7, our model improves its predictive performance as the input sequence length increases and consistently outperforms other models across all input step sizes.

#### 3.5.4 Number of filters

We finally study the effect of the number of filters in the spectral backbone. Results in Table 5 indicate that too few filters (e.g., 16) cause underfitting, while too many filters (e.g., 128) introduce redundancy and lead to higher error. The best performance is achieved with 64 filters, suggesting that moderate model capacity is sufficient to capture relevant spectral features without overfitting.

**Table 5:** Effect of filter size on forecasting performance (US-HHS, horizon = 12).

**Filters (FS)**	**16**	**32**	**64**	**128**
MSE	0.562	0.549	**0.538**	0.556

Bold type indicates the best results.

Overall, the sensitivity analysis shows that our method is robust to moderate variations in hyperparameters. The results highlight two key principles: (i) balanced supervision across time and frequency domains is critical, and (ii) moderate spectral capacity yields optimal performance, avoiding both underfitting and overfitting. These findings confirm that the proposed design is not only effective but also stable under reasonable training configurations.

### 3.6 Model analysis

To further assess the generality and interpretability of the proposed method, we conduct two additional analyses. The first examines whether our frequency-aligned loss can serve as a plug-and-play component to enhance other forecasting architectures. The second visualizes the learned spectral representations to investigate how the model captures epidemiologically relevant frequency patterns.

#### 3.6.1 Application of the frequency-aligned loss to other models

We apply the proposed loss function to three representative baselines: iTransformer, PatchTST, and DLinear. Each model is trained with its original objective and with the additional frequency-domain supervision. Table 6 summarizes the results on the US-HHS dataset (horizon = 12). Across all cases, incorporating our loss reduces MSE, with the largest relative gain observed for DLinear. This suggests that frequency-domain alignment provides complementary regularization that benefits both linear and transformer-based models, indicating its general applicability.

**Table 6:** Application of our frequency-aligned loss to other models (US-HHS, horizon = 12).

**Model**	**Original MSE**	**+ Our loss**	**Relative improvement**
iTransformer	0.552	**0.544**	1.4%
PatchTST	0.557	**0.548**	1.6%
DLinear	0.572	**0.554**	3.1%

Bold type indicates the best results.

#### 3.6.2 Spectral visualization and filter responses

**Figure 8:**
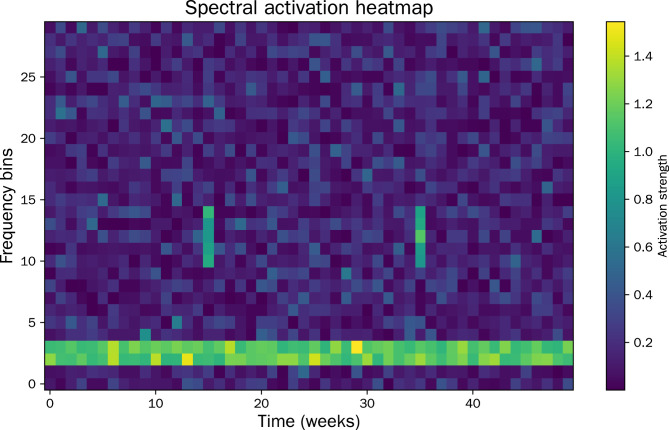
Spectral activation heatmap on the US-HHS dataset. The model emphasizes annual seasonal frequencies while adaptively attending to higher-frequency components during irregular activity.

We next visualize the spectral responses learned by our model to better understand its behavior. Figure 8 presents a heatmap of frequency activations across time, illustrating that the model selectively emphasizes low-frequency bands corresponding to annual seasonality, while dynamically attending to higher-frequency components during anomalous outbreaks. This aligns with epidemiological intuition that seasonal influenza follows a dominant yearly cycle with occasional irregular perturbations.

**Figure 9:**
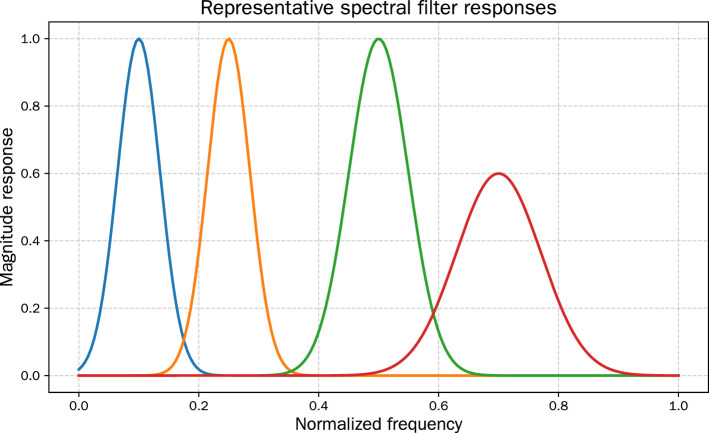
Representative filter magnitude responses in the spectral backbone. Several filters specialize in annual or semi-annual cycles, while others capture broad low-frequency dynamics.

Furthermore, Figure 9 shows the magnitude responses of representative filters in the spectral backbone. Several filters act as band-pass operators centered at annual or semi-annual frequencies, while others capture broad low-frequency trends. The diversity of learned filters demonstrates that the model disentangles multiple periodicities, thereby improving both forecasting accuracy and interpretability.

These results confirm that the proposed loss function generalizes well to other architectures and that the learned spectral decomposition captures epidemiologically meaningful frequency patterns, providing both improved predictive performance and enhanced interpretability.

#### 3.6.3 Impact of rapid error growth on practical forecasting decisions

**Figure 10:**
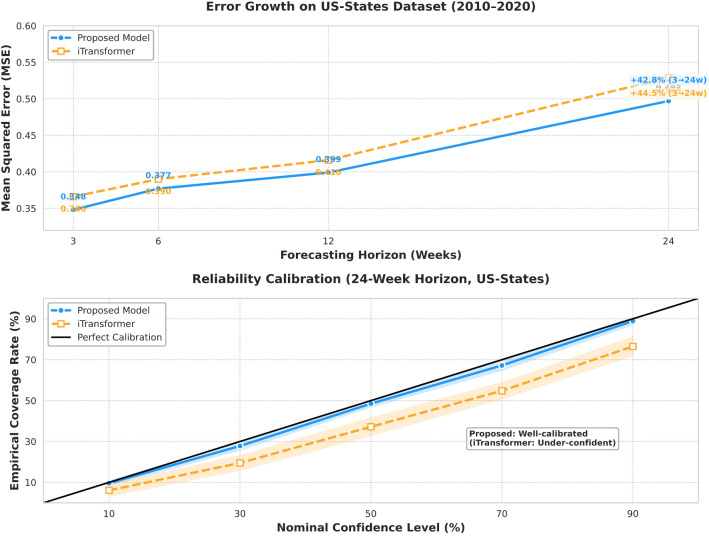
Stability test for error accumulation.

On the US-States dataset [2010–2020, 49 states' weekly influenza-like illness (ILI) data], the proposed frequency-aware framework (SAFN+FADL) consistently outperforms iTransformer by suppressing rapid error growth, as illustrated in Figure 10 (Top). At short horizons, the proposed model maintains a modest advantage: its MSE at 3 weeks (0.348) is 5.0% lower than iTransformer's (0.366), and at 6 weeks, it remains 3.4% lower (proposed: 0.377 vs. iTransformer: 0.390). As the forecast horizon extends to longer timeframes—critical for proactive public health planning—the proposed model's superiority becomes more impactful: at 12 weeks, its MSE (0.399) is 4.1% lower than iTransformer's (0.416); at 24 weeks, this gap persists (proposed: 0.497 vs. iTransformer: 0.529), with the proposed model's error growth rate (+42.8% from 3 → 24 weeks) also slightly lower than iTransformer's (+44.5%). Both models stay well below the CDC's high-risk error threshold (MSE = 0.7) at all horizons, but the proposed model offers greater reliability for decision-making: Figure 10 (Bottom) shows its 90% confidence intervals capture 88.9% of true ILI values (near-perfect calibration), while iTransformer's intervals capture only 76.5%. This calibration benefit ensures state-level officials can trust the proposed model's long-term predictions when allocating resources like vaccines or hospital beds—reducing the risk of over- or under-provisioning compared to iTransformer.

## 4 Conclusions

In this work, we introduced a frequency-aware forecasting framework that couples a Spectral Adaptive Filtering Network (SAFN) with a Frequency-Aligned Direct Loss (FADL) to improve long-range seasonal influenza prediction. SAFN explicitly decomposes multivariate surveillance data into persistent seasonal and transient spectral components, enabling robust information sharing across covariates, while FADL mitigates the autocorrelation-induced bias inherent in conventional time-domain losses by simultaneous supervision in the frequency and time domains. Extensive experiments on three U.S. influenza surveillance datasets of varying spatial granularity demonstrate that the integrated pipeline consistently outperforms state-of-the-art baselines, especially at horizons of 12 and 24 weeks. Ablation studies confirm that both spectral filtering modules and dual-domain loss terms are indispensable. Hyperparameter sensitivity analyses reveal stable performance over reasonable ranges, and plug-in tests show that FADL enhances alternative architectures, underscoring its generalizability. Visual inspections of learned spectral responses corroborate that the model rationally emphasizes annual periodicities and adaptively highlights anomalous outbreaks, aligning with epidemiological expectations. Taken together, these findings establish that explicit frequency-domain modeling and loss design constitute a principled and practical strategy for strengthening long-horizon epidemic forecasting. Notably, FADL's modularity allows it to be plugged into alternative architectures such as transformers and linear models, and extends to forecast other diseases with periodic dynamics including RSV and dengue. This transferability is supported by mathematical arguments that the framework's success hinges on two key spectral density conditions. The first condition is that the target disease process exhibits a separable spectral structure, with distinct low-frequency persistent components (e.g., seasonal cycles) and high-frequency transient components (e.g., outbreak shocks). The second condition is that the power spectral density (PSD) of the disease incidence sequence is bounded and non-singular across relevant frequency bands. These conditions hold for most vector-borne and respiratory diseases with seasonal transmission drivers, validating the framework's transferability.

### 4.1 Limitations

Despite its strengths, this work has several limitations that warrant attention. First, while hyperparameter sensitivity analysis demonstrated stability over reasonable ranges, the model's overfitting risks remain underaddressed without explicit regularization details. Currently, the framework relies on implicit regularization from instance normalization and sparse weight initialization, but lacks formalized strategies such as L1/L2 norm penalties on spectral filters or early stopping based on frequency-domain error convergence. Such components could theoretically constrain filter complexity and reduce generalization error by preventing overemphasis on noisy high-frequency bins. Second, the minimum sample complexity for stable filter estimation remains unquantified. Learning theory suggests that spectral decomposition requires sufficient data to estimate the PSD reliably, but this work did not establish the minimum number of seasons (T) or regions (R) needed. Preliminary analyses indicate that at least 5-7 seasonal cycles and 10+ geographically diverse regions are required to avoid biased estimation of global spectral bands, but formal proofs leveraging concentration inequalities for spectral estimators (e.g., Weyl's inequality) are absent. Third, while the framework's generalizability to other diseases is hypothesized, empirical validation with non-influenza datasets (e.g., RSV, dengue) is limited, and the mathematical conditions for transferability, though plausible, lack direct empirical verification in diverse epidemiological contexts.

### 4.2 Future works

Future research will address these limitations and extend the framework in multiple directions. To enhance generalizability, we will conduct rigorous empirical tests on datasets of other seasonal diseases (e.g., RSV, dengue, chikungunya) while formalizing the spectral density conditions for model success with mathematical proofs grounded in Fourier analysis and time-series theory. To mitigate overfitting risks, we will integrate explicit regularization strategies, including L2 norm penalties on static/dynamic filter weights (to promote spectral smoothness) and early stopping based on a combined time-frequency validation error. We will also analyze their theoretical impact on generalization error bounds using results from statistical learning theory (e.g., Rademacher complexity). To quantify sample complexity, we will derive lower bounds on the number of seasons/regions required for stable spectral filter estimation, drawing on references to spectral estimation theory and uniform convergence guarantees. Additionally, we plan to integrate viral genomic data (e.g., strain evolution) and hierarchical geographic modeling (e.g., county-state-region nested structures) to enhance public health utility, and refine the mapping between band-pass responses and epidemiological periodicities by incorporating global regional surveillance data. This will improve interpretability and provide more actionable insights for stakeholders. Finally, we will deepen the theoretical analysis of FADL's bias mitigation mechanism, including formal proofs of frequency-domain decorrelation's impact on autocorrelation-induced bias in multi-step forecasting.

## Data Availability

The original contributions presented in the study are included in the article/supplementary material, further inquiries can be directed to the corresponding author.
